# Non-association of IL-12 +1188 and IFN-γ +874 Polymorphisms with Cytokines Serum Level in Occult HBV Infected Patients

**DOI:** 10.4103/1319-3767.74461

**Published:** 2011

**Authors:** Mohammad K. Arababadi, Ali A. Pourfathollah, Abdollah Jafarzadeh, Gholamhossein Hassanshahi, Saeed Daneshmandi, Ali Shamsizadeh, Derek Kennedy

**Affiliations:** 1 Department of Microbiology, Hematology and Immunology, Faculty of Medicine, Rafsanjan University of Medical Sciences, Rafsanjan, Iran; 2 Molecular- Medicine Research Center, Rafsanjan University of Medical Sciences, Rafsanjan, Iran; 3 Department of Immunology, School of Medical Sciences, Tarbiat Modares University, Tehran, Iran; 4 Department of Physiology, Faculty of Medicine, Rafsanjan University of Medical Sciences, Rafsanjan, Iran; 5 School of Biomolecular and Biomedical Science, Eskitis Institute for Cell and Molecular Therapies, Griffi th University Nathan, Queensland, Australia

**Keywords:** IL-12, IFN-γ, occult hepatitis B infection, polymorphism

## Abstract

**Background/Aim:**

Occult hepatitis B infection (OBI) is identified as a form of hepatitis in which despite the absence of detectable HBsAg, HBV-DNA is observed in peripheral blood of patients. The main aim of this study has been to investigate the association between polymorphisms in +874 of IFN-γ and +1188 of IL-12 with their serum level in patients suffering from OBI.

**Materials and Methods:**

In this experimental study, plasma samples of 3700 blood donors were tested for the presence of hepatitis B surface antigen (HBsAg) and anti-HBc by ELISA. The HBsAg^-^/anti-HBc^+^ samples were selected and screened for HBV-DNA by PCR. HBV-DNA positive samples were assigned as OBI cases and ARMS-PCR techniques were performed to examine the two known polymorphisms within IL-12 and IFN-γ. In addition, the serum levels of IL-12 and IFN-γ were also determined by ELISA.

**Results:**

Results of this study demonstrated that, 352 (9.5%) out of 3700 blood samples were HBsAg^-^/anti-HBc^+^ and HBV-DNA was detected in 57/352 (16.1%) of HBsAg^-^/anti-HBc^+^ samples. Our results showed that groups showed significant difference in CC allele of +1188 region of IL-12 and no difference was observed in the other evaluated genes. Our results also showed that the alleles of +1188 region of IL-12 and alleles of +874 of IFN-γ were also not associated with serum level of cytokines.

**Conclusion:**

According to the results of this study, it may be concluded that the polymorphisms in +1188 region of IL-12 and +874 region of IFN-γ would not affect the expression of both cytokines at serum level in OBI patients.

Occult HBV infection (OBI) is a clinical form of hepatitis B in which there are undetectable amounts of HBsAg in patient’s serum, despite being positive for HBV-DNA.[1,2] This type of hepatitis is one of the main challenges for blood transfusion services. Despite appropriate screening of all donated blood and blood components for HBsAg, some cases of post-transfusion hepatitis B are reported.[3] The majority of post transfusion hepatitis B infections are caused by OBI[4] which we found in our previous investigations in Isfahan and Kerman, the two central provinces of Iran.[4,5] The mechanisms responsible for progression of OBI are yet to be clarified; however, some investigators have suggested that genetic and immunological parameters may play a significant role in the resistance of some individuals and sensitivity of other patients.[6,7] Cytokines play important role in initiating and maintaining an appropriate immune response to viral infections.[8] IL-12 and IFN-γ are two of the main cytokines involved in the induction of cellular immunity against viral infections, especially HBV.[8] Therefore, genetic factors that affect expression of cytokines that regulate and initiate the immune system may in turn reduce the ability of the immune system to elicit a response against viral infections.[9] Previous studies showed that the polymorphisms within IL-12[10] and IFN-γ[11,12] have been correlated with HBV infection. Therefore, the aim of this study has been to investigate the relationship between OBI and functional polymorphisms in IL-12 (+1188) and IFN-γ (+874), as well as serum levels of these cytokines. An additional aim of this study was to find out the association between these polymorphisms and serum levels of IL-12 and IFN-γ as the main cytokines involved in cellular immunity.[8]

## MATERIALS AND METHODS

### Patients

Peripheral blood samples were collected from 3700 volunteer blood donors of the Rafsanjan Blood Transfusion Services (Kerman, Iran) and placed in EDTA pre-coated 5.5 ml tubes. The samples were centrifuged at 370 × g for 4 min and the sera collected. All sera were separated within 24 h of collection. If needed, serum samples were stored at –20°C for a maximum of two months or at -70°C, where longer storage times were required, for further processing. For analysis of polymorphisms, a 2 ml sample was collected from patients with OBI (57 cases) and one hundred healthy controls (HBsAg^-^/HBV-DNA^-^/anti-HBc^+^). The study protocol was approved by the ethical committee of Rafsanjan University of Medical Sciences.

All the participants of this study filled out and signed the informed consent form which was designed based on the aims and objectives of the study.

### Detection of serological HBV markers

HBsAg screening tests were performed by enzyme linked immuno-sorbent assay (ELISA) (Behring, Germany). Anti-HBc screening tests were also performed by a manual microplate enzyme immunoassay using an anti-HBc commercial kit (RADIM, Italy). The present method is based on a competitive enzyme immunoassay (EIA). All of the samples were also screened by ELISA (RADIM, Italy) for possible HCV, HIV and HTLV-1 infections.

### HBV- DNA extraction from plasma samples

Viral DNA was purified from 200 *µ*l of plasma samples. Briefly, each plasma sample was incubated at 72°C for 10 min and then cooled down to 4°C for 5 min in 200 *µ*l proteinase K (200 *µ*g/ml). Following phenol/chloroform extraction (1:1), the viral DNA was precipitated with ethanol and the pellet was re-dissolved in DNase free, deionized water and stored at –20°C for further use.

### HBV-DNA PCR and gel electrophoresis

PCR was carried out in a 25 *µ*l mixture containing 10 mM tris-HCl (pH 8.3), 50 mM KCl, 1.5 mM MgCl2, 0.01 % gelatin, 5 units recombinant *Taq* DNA polymerase, 200 *µ*M of each dNTPs, 0.6 *µ*M of each primer, and 5 *µ*l of the DNA extracted from 200 *µ*l of plasma. The sequences of all primers used in this study are shown in Table 1. For HBV analysis, the primers are designed to amplify a 500bp amplicon of the surface antigen or S gene of HBV genome. Fast temperature cycling was performed. PCR amplification was performed using the touch down method which included one cycle of 93°C for 60 s, 60°C for 20 s and 72°C for 40 s, then 5 cycles of 93°C for 20 s, 60°C to 56°C for 20 s and 72°C for 40 s followed by 30 cycles of 93°C for 20 s, 55°C for 20 s and 72°C for 40 s. HBV genomic DNA provided by the Cinnagen company (Iran) was used as positive control. For the analysis of the PCR amplification, 10 *µ*l of the amplified DNA were run on a 2% agarose gel after addition of 4 *µ*l of loading buffer. The presence of a 500 bp fragment indicated positive result. In parallel with samples, a 100 bp DNA ladder was also run on the gels to estimate the molecular weight of DNA fragments in the gel.

**Table 1:** Sequences of primers and size fragment of PCR production

Genes	Primers	Product size (bp)	References
S gene (HBV)	F: TCGTGGTGGACTTCTCTC	500	[1]
	R: ACAGTGGGGGAAAGCCC		
IL-12	IL-12 +1188 Common (F): GACACAACGGAATAGACC	116	[10]
	IL-12 +1188 A: AATGAGCATTTAGCATCT		
	IL-12 +1188 C: AATGAGCATTTAGCATCG		
Control of IL-12 amplification (beta globulin)	F: TGCCAAGTGGAGCACCCAA	796	[10]
	R: GCATCTTGCTCTGTGCAGAT		
IFN-γ	IFN-γ +874 Common (F): GACACAACGGAATAGACC	262	[9]
	IFN-γ +874 A: AATGAGCATTTAGCATCT		
	IFN-γ +874 C: AATGAGCATTTAGCATCG		
Control of IFN-γ amplification (beta globulin)	F: ACACAACTGTGTTCACTAGC	100	[9]
	R: CAACTTCATCCACGTTCACC		

### Genomic DNA extraction

Peripheral blood was collected on EDTA and genomic DNA was extracted by a commercial kit (Bioneer, Korea) using the recommended procedures. Extracted DNA was aliquoted (for each sample) and stored at -20°C for further use.

### Detection of polymorphisms

Polymorphisms at position of +874 region of IFN-γ and the +1188 region of IL-12 genes were identified using ARMS-PCR with specific primers of interest [Table 1].

PCR was performed in a volume of 50 *µ*l containing 250 ng of DNA templates, 200 *µ*M of each dNTP (Cinnagen-Iran), 0.5 U *Taq* DNA polymerase (Cinnagen-Iran), 1× PCR buffer (Cinnagen- Iran), 3 mM MgCl2, and 5 pM of each specific primer [Table 1]. The PCR condition was an initial denaturation at 95°C for 5 min, followed by 35 cycles of melting at 95°C for 50 s, annealing at 53°C (for IFN-) and 58.8°C (for IL-12) for 50 s, and extension at 72°C for 5 s, with a final extension step of 5 min at 72°C using a Mastercycler thermal cycler (Eppendorf, Germany). The expected PCR products were 262bp for IFN-γ and 116bp for IL-12. The products were run on a 2% agarose gel (Cinnagen-Iran) and scored over a UV transilluminator after staining with ethidium bromide.

### Cytokine level assay

The serum levels of IL-12 and IFN-γ were measured by ELISA (eBioscience, ESP) in patients and healthy controls immediately after blood collection. Assays were performed according to the manufacturer’s protocols. The sensitivity of the kit was ±2 pg/ml and inter- and intra-assay assessments of reliability of the kit were conducted.

## RESULTS

This study was performed on 3700 blood samples collected from the Rafsanjan blood transfusion services. All of samples were found to be negative for HBsAg and HCV, HTLV-1 and HIV antibodies. Out of 3700 samples, 352 (9.5%) cases were positive for anti-HBc and HBV-DNA was detected in 57/352 (16.1% of HBsAg negative but anti-HBc positive) of those samples. Results of this study indicated that 16.1 % of HBsAg negative but anti-HBc positive samples had detectable HBV-DNA which is 1.54% (57/3700) of total collected samples.

The mean age in patients and control groups was 28 ± 6 and 28 ± 8, respectively and there was no significant difference in age between the two groups [Table 2]. Three (3%) of the control group members were females and 97 (97%) were males while two of patients (3.5%) were females and 55 (96.5%) were males. There was no significant difference regarding the age of the control versus patient groups. In addition, analysis of socio-economic conditions showed that there was also no significant difference between the patient and control groups [Table 2]. In this series of experiments, we found that 20 (35.1%) patients had AA allele in the +1188 region of IL-12 whereas 36 (36%) of control cases showed this allele. The difference was not significant between the groups (*P*>0.90). In respect to the AC allele, our results showed that 37 (64.9%) of OBI patients and 54 (54%) of control cases contained the AC allele in the +1188 region of IL-12 indicating that there were no significant differences between the groups (*P*>0.24). Only 10 cases of control group and none of the patients, within the test group, had the CC alleles which represents a significant difference between the patient and control groups for this allele (*P*<0.033) [Table 3].

**Table 2:** Demographic and socioeconomic conditions of occult hepatitis B virus-infected patients and controls

Variant	Healthy control	Patient
Age	28 ± 8	28 ± 6
Sex		
Female	3 (3)	2 (3.5)
Male	97 (97.8)	55 (96.5)
Socio-economic status		
Weak	22 (22)	12 (21)
Medium	47 (47)	28 (49)
High	31 (31)	17 (30)

Figures in parentheses are in percentage

**Table 3:** Indicates polymorphisms in +1188 region of IL-12 gene in OBI patients and controls

Polymorphisms / condition	AA *n* (%)	AC *n* (%)	CC *n* (%)	Total *n* (%)
Patients	20	37	0	57
	35.1	64.9	0	100
Healthy control	36	54	10	100
	36	54%	10	100
*P* value	>0.90	>0.24	<0.033	

OBI: Occult hepatitis B infection

Our results also showed 18 (31.5%) patients with the AA allele, 25 (43.8%) with AT and 14 (24.7%) with the TT allele at the +874 region of IFN-γ. In control group 28 (28%) cases had AA allele, 47 (47%) AT and 25 (25%) TT in +874 region of IFN-γ which was not a significant difference to the patient group (*P*>0.1) [Table 4].

**Table 4:** Polymorphisms in +874 region of IFN-γ gene in OBI patients and controls

Polymorphisms / condition	AA *n* (%)	AT *n* (%)	TT *n* (%)	Total *n* (%)
Patients	18	25	14	57
	31.5	43.8	24.7	100
Healthy control	28	47	25	100
	28	47	25	100
*P* value	>0.1	>0.1	>0.1	

OBI: Occult hepatitis B infection

**Figure 1:**
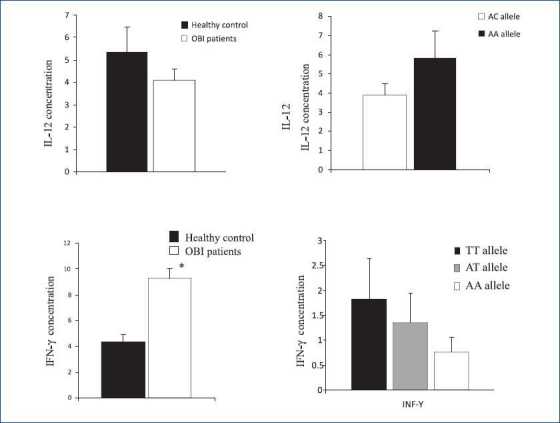
Serum levels of IL-12 and IFN-γ genes in OBI and their correlation with the polymorphisms within IL-12 and IFN- patients and healthy controls. As it is obvious in figure, no signifi cant differences were seen regarding serum levels of IL-12 in OBI patients in comparison with controls (a) (*P*>0.1) while the serum was increased (c) (levels of IFN-γ, *P*<0.001). No significant differences were seen between the polymorphisms within IL-12 (b) (*P*>0.1) and IFN-γ (d) (*P*>0.1) genes and their serum levels. Data are shown as mean ± SE

The results of this study showed that serum levels of IL-12 were 4.06 ± 0.53 and 5.34 ± 1.11 pg/ml in OBI patients and healthy controls, respectively (*P*>0.1) [Figure 1]. Our results have also not shown any difference in serum levels of IL-12 between the OBI patients with AA and AC alleles (*P*>0.1) [Figure 1].

Evaluation of serum levels of IFN-γ showed that concentrations of IFN-γ were significantly higher in OBI patients (9.26 ± 0.8 pg/ml) in comparison to the control group (4.2 ± 0.6 pg/ml) (*P*<0.001) [Figure 1]. The results of this study also showed that the serum levels of IFN-γ were 1.83 ± 0.8, 1.34 ± 0.6 and 0.75 ± 0.3 pg/ml for the TT, AT and AA alleles, respectively [Figure 1].

## DISCUSSION

During viral infections, the expression pattern of cytokines is changed and IL-12 and IFN-γ are increased.[13] Studies showed that polymorphisms in specific regions of these cytokines also influence the expression pattern of cytokines.[14] For example, the +1188 polymorphism of P40 subunit of IL-12 gene is believed to be involved in regulation of IL-12 expression.[10] It is not clear which mechanisms are being affected in OBI patients that make them unable to completely recover from viral infections. However, it seems that cytokines play key roles in clearance of HBV and several studies indicated that NK cells and cytotoxic T cells (the two important cells in cellular immunity) depend on cytokine balance to attain optimal function.[15,16] Therefore, this study aimed to examine the association of polymorphisms of IL-12 and IFN-γ with their serum level, as these represent the regulatory cytokines of cellular immunity. In our previous studies, we reported OBI in Iranian blood donors.[4,5] In the current study, we could not find a significant association between the +874 region of IFN-γ and OBI, but our results are in contrast to the findings of Zhu *et al*. in intra-uterine HBV.[14] They showed that there was a significant difference between AA allele and intra-uterine HBV infection. Hui *et al*. also reported that there is a relationship between AT alleles of this region in IFN-γ and intra-uterine HBV[10] which is in contrast with our results. In contrast with our own results, most studies indicated that the AA allele is related to hepatitis B. The three following reasons could partially describe this discrepancy: 1. Our patients are different in race and genetic origin. This is plausible because most of the reports originate from the South-East Asian countries and the race and genetic composition of this population may vary from our population, this may suggest that additional genetic factors influence cytokine responses to viral infection in these regions. 2. The two clinical and immunological aspects of OBI could be quite different from other clinical presentations of hepatitis B and therefore may not be comparable to chronic hepatitis B infection. A separate study in Chinese patients suffering from chronic hepatitis B showed that the AA allele is more frequent in these patients.[17]

We also showed that the serum level of IFN-γ is lower in patients with AA allele but this was not statistically significant and it seems that with more patients or *in vitro* mitogen activation it would possibly become significant. On the other hand, the serum level of this cytokine in OBI patients was elevated, thus, it could be concluded that these patients have no apparent difficulty in production of IFN-γ. In fact, the AA allele in +874 of IFN-γ could be considered as a risk factor of eradication of HBV by the immune system.

In contrast to the IFN-γ, our results showed that there was a significant correlation between the CC allele of +1188 region in IL-12 in OBI and the results of this study showed that CC allele of IL-12 correlated with OBI. Previous studies showed that CC alleles of +1188 region of IL-12 are related to low expression of IL-12 by immune cells.[18] Our results also showed that the serum levels of IL-12 were not increased in patients with OBI; hence, it could be concluded that these patients are unable to express high enough concentrations of this cytokine to facilitate HBV clearance. However, Park *et al*. were unable unable to find a clear relationship between these alleles and HBV infection in the Korea population.[10] Assessment of IL-12 polymorphisms and HBV was not the focus of the investigators; hence, there was not much information in the data base for comparison. In addition, some of the available information are the results of studies performed by investigators within the South-East Asian regions, and as indicated earlier could represent a population that is genotypically quite different from our country. Due to the ethnic differences between the populations, more studies are needed in our country to accurately define the relationship between OBI and these polymorphisms.

Our results did not show a significant difference in IL-12 serum levels in AA and AC genotypes. However, based on the fact that, in our observations, the OBI patients did not produce sufficient levels of this cytokine; the association of alleles in +1188 region of IL-12 gene with its serum protein levels may become significant with more patients and *in vitro* mitogen activation.

Finally, due to the complexity of OBI, other aspects of the disease need to be examined and it is recommended to study the expressions and polymorphisms of other important related cytokines and their receptors in OBI patients as a future work.
